# Supplementary material for: Genome-Wide Association Analysis of Boar Semen Traits Based on Computer-Assisted Semen Analysis and Flow Cytometry
Source: Animals (Basel). 2024 Dec 26;15(1):26. doi: 10.3390/ani15010026 (PMC11718925; doi:10.3390/ani15010026)
Supplement: Supplementary file 1 [file animals-15-00026-s001.zip › animals-3343041-supplementary.pdf]

Supplementary material

Supplementary Figure S1.

Format: DOC

Title: Phenotypic distribution of semen traits data in Duroc breed, including MOT (a), DEN (b), ABN(c), AIR(d), MMP (e), ROS (f).

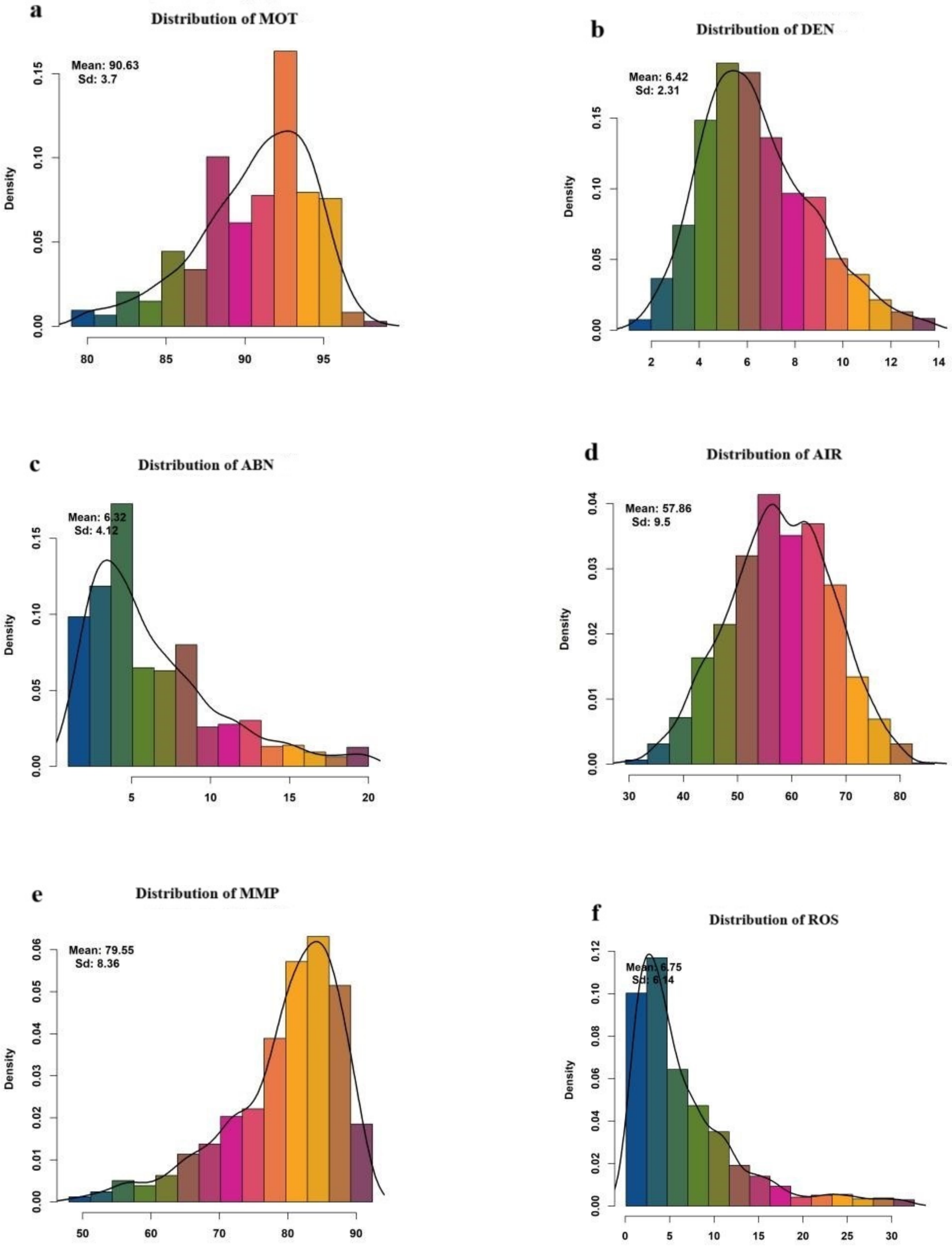

Supplementary Figure S2.

Format: DOC

Title: SNPs density across genome in Duroc pigs.

Description: SNPs density on different chromosomes, except sex chromosomes for Duroc pigs. The whole genome was divided into 1 Mb windows and marked with different colors based on the marker density of the window.

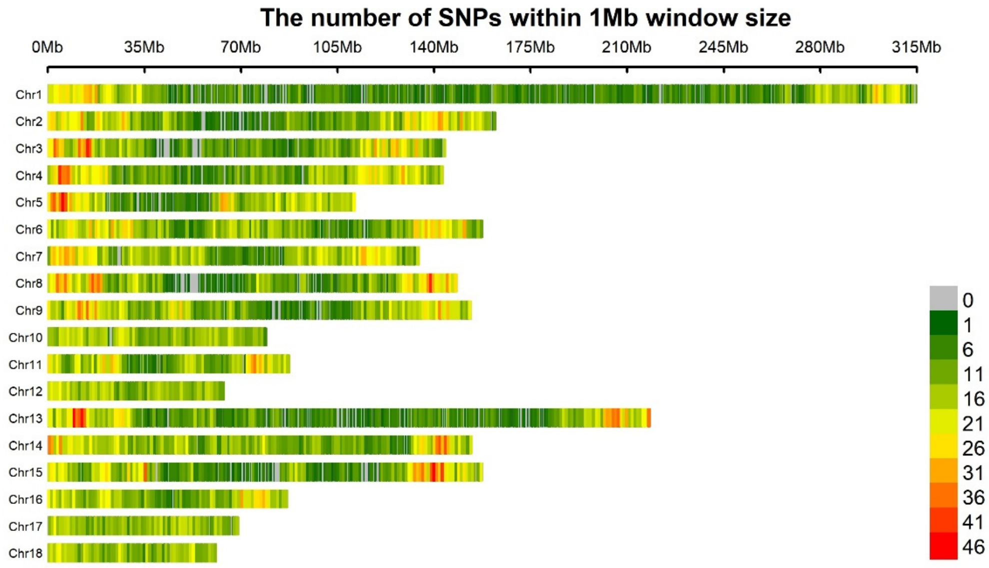

Supplementary Figure S3.

Format: DOC

Title: The first two principal components are plotted to display the population structure Duroc breed.

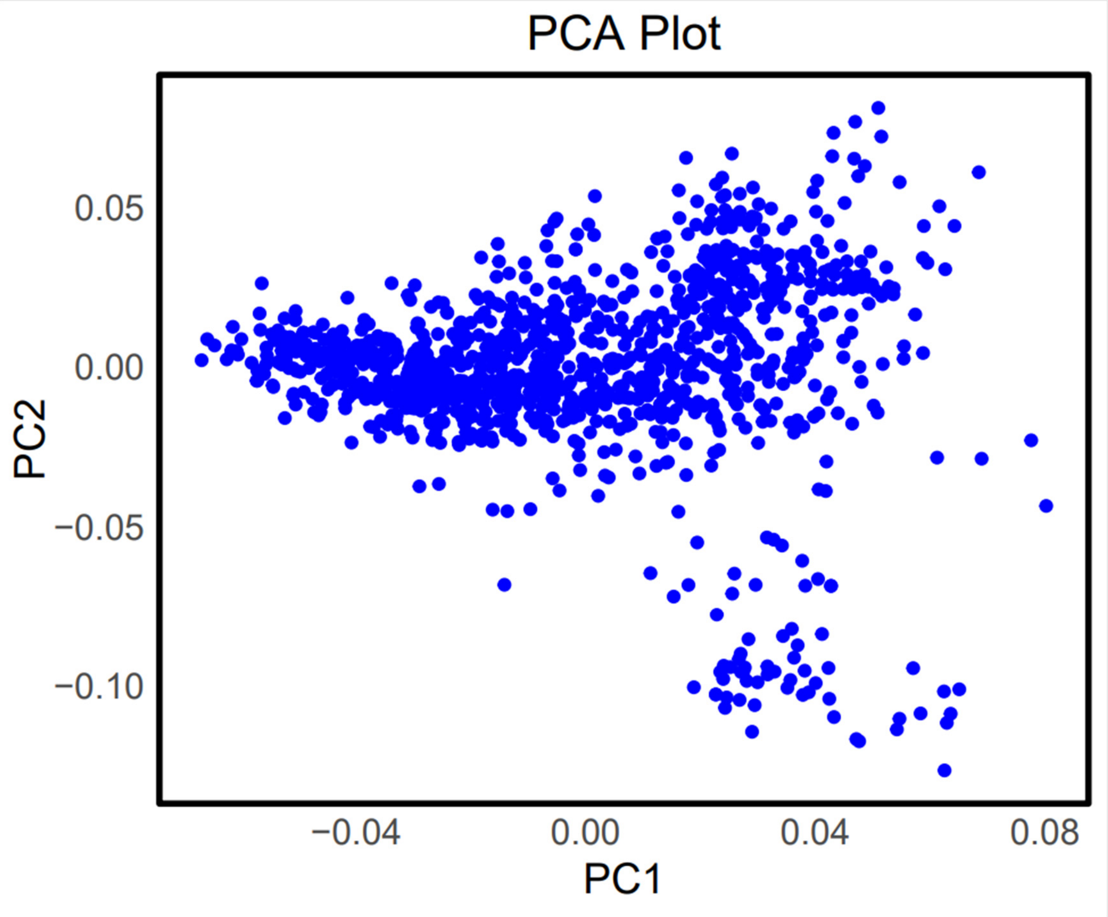

Supplementary Figure S4.

Format: DOC

Title: The linkage disequilibrium decay in populations of Landrace.

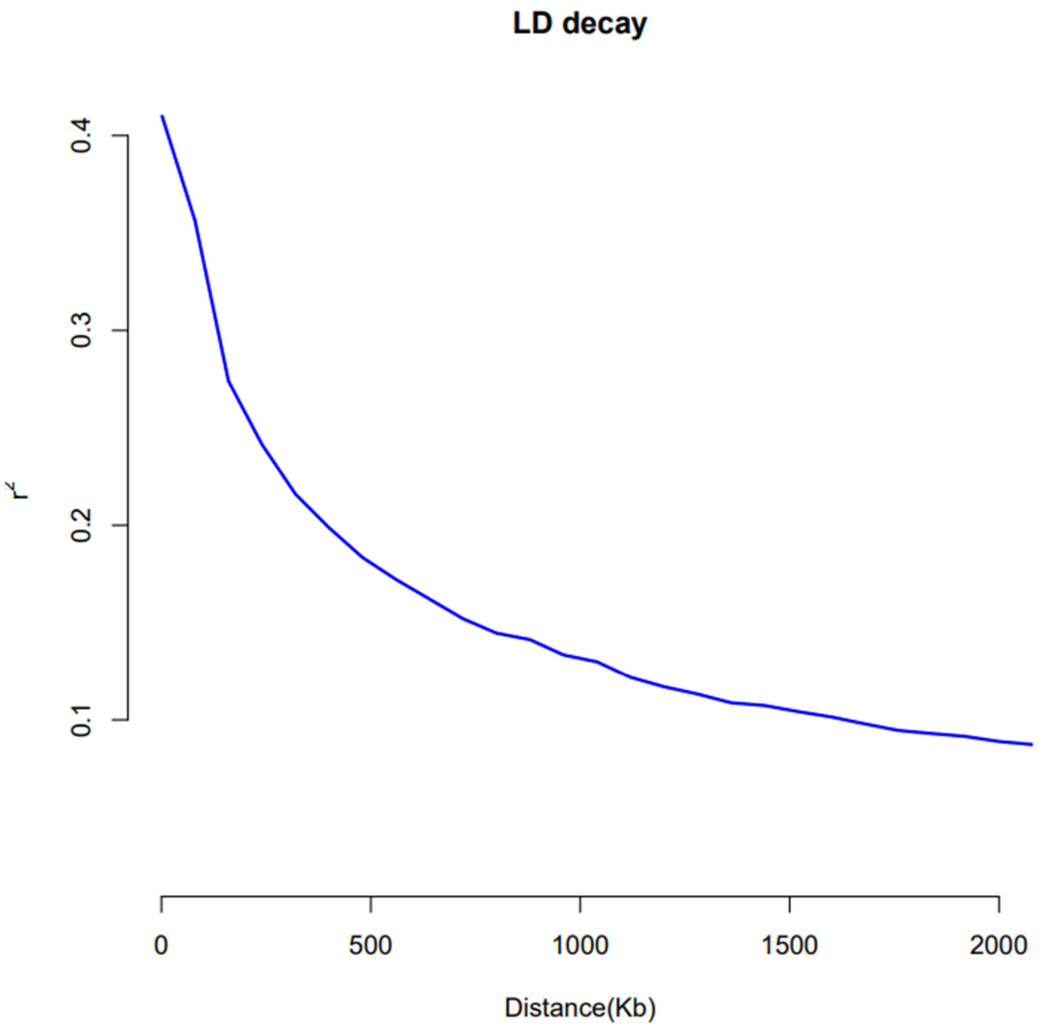

Supplementary Table S1

Format: DOC

Title:Significant and potential SNP in genome-wide association analysis of DEN.

SNP	Chr	Location （bp）	P-value	Gene names	Bilateral genes	Distance	Method
DRGA0000548	1	34548111	3.10E-05	--	--	--	I,II
					PTPRK	+145257	
ASGA0002287	1	34895943	2.63E-05	PTPRK	--	--	I,II
					THEMIS	+623927	
MARC0031963	1	105818705	2.25E-05	TXNL1	--	--	I,II
					WDR7	+217324	
INRA0003610	1	105751534	1.88E-05	TXNL1	--	--	I,II
					WDR7	+284495	
WU_10.2_6_3178374	6	2916445	2.10E-06	--	FOXF1	-318786	I,II
					IRF8	+125200	
WU_10.2_6_3241813	6	2889277	2.17E-05	--	FOXF1	-291618	I,II
					IRF8	+152368	
H3GA0054036	8	4676256	1.83E-05	--	JAKMIP1	-64052	I,II
					CRMP1	+57944	
ALGA0104245	9	65384410	1.91E-05	LRRN2	MDM4	-164668	I,II
					NFASC	+117849	
**WU_10.2_14_4278229**	**14**	**4243667**	**4.33E-07**	**SLC18A1**	**LPL**	**-107853**	**I,II**
					ATP6V1B2	+45672	
**MARC0028432**	**14**	**4271553**	**4.47E-07**	**SLC18A1**	**LPL**	**-135739**	**I,II**

					**ATP6V1B2**	**+17786**	
**MARC0017786**	**14**	**4323106**	**4.47E-07**	**ATP6V1B2**	**SLC18A1**	**-47863**	**I,II**
					LZTS1	+23374	
MARC0011591	14	123359191	1.87E-05	TCF7L2	VTI1A	-143253	I,II
					U6	+30103	
MARC0032193	16	23357087	3.09E-05	U6	GDNF	-390551	I,II
					EGFLAM	+152404	
INRA0051369	16	28118638	1.28E-05	PAIP1	C5orf34	-24186	I,II
					NNT	+38680	
DRGA0016090	16	33372038	3.44E-06	ARL15	NDUFS4	-384210	I,II
					HSPB3	+344588	
ALGA0121589	16	23789565	1.90E-05	LIFR	EGFLAM	-182641	I,II
					OSMR	+252002	
ALGA0120801	16	27608306	1.86E-05	U6	CCDC152	-69641	I,II
					ZNF131	+205691	
ASGA0106422	16	27618318	1.86E-05	U6	CCDC152	-79653	I,II
					ZNF131	+195679	
WU_10.2_16_29287377	16	27743279	1.86E-05	--	U6	-116432	I,II
					ZNF131	+70718	
MARC0020433	16	31262432	2.72E-05	--	ISL1	-288525	I,II
					--	--	
ALGA0090125	16	32246012	1.65E-05	ITGA1	PELO	-103978	I,II
					--	--	
ALGA0090122	16	32220183	1.55E-05	PELO	ITGA1	-90571	I,II
					ITGA2	+116109	

Supplementary Table S1.Cont.

SNP	Chr	Location（bp）	P-value	Gene names	Bilateral genes	Distance	Method
ALGA0090184	16	33467573	2.64E-06	ARL15	NDUFS4	-479745	I,II
					HSPB3	+249053	
MARC0103451	16	33493718	3.77E-06	ARL15	--	--	I,II
					HSPB3	+222908	
ASGA0072998	16	33589982	7.05E-06	--	ARL15	-65681	I,II
					HSPB3	+126644	
ASGA0073002	16	33636702	7.33E-06	--	ARL15	-112401	I,II
					HSPB3	+79924	
ALGA0090209	16	33649916	1.45E-05	--	ARL15	-125615	I,II
					HSPB3	+66710	
WU_10.2_16_35775083	16	33703674	1.41E-05	HSPB3	ARL15	-179373	I,II
					SNX18	+73072	
WU_10.2_16_35829257	16	33757844	1.84E-05	SNX18	HSPB3	-38826	I,II
					ESM1	+426413	
MARC0074483	16	35444990	1.54E-05	--	ANKRD55	-114200	I,II
					U6	+81123	
WU_10.2_16_37847318	16	35580651	1.54E-05	U3	U6	-54431	I,II
					MAP3K1	+291480	
ASGA0073072	16	35263239	2.30E-05	ANKRD55	IL6ST	-71019	I,II
					U6	+262874	
WU_10.2_16_49341867	16	45722803	1.80E-05	--	CD180	-134737	I,II
					--	--	

Supplementary Table S1.Cont.

SNP	Chr	Location（bp）	P-value	Gene names	Bilateral genes	Distance	Method
ALGA0090628	16	45790346	1.98E-05	--	CD180<br>--	-202280<br>--	I,II
WU_10.2_16_50400065	16	46638062	9.79E-06	--	PIK3R1<br>--	-114453<br>--	I,II
WU_10.2_16_50618846	16	46754289	1.22E-05	--	PIK3R1<br>--	-230680<br>--	I,II
MARC0099891	6	94684952	2.71E-05	RRAGC	--<br>MYCBP	--<br>+120688	I,II
WU_10.2_14_134255064	14	123012035	1.96E-05	VTI1A	ZDHHC6<br>TCF7L2	-163456<br>+331377	I,II
DRGA0016110	16	35517389	1.54E-05	U6	ANKRD55<br>U3	-186599<br>+60470	I,II
ALGA0090190	16	33515233	4.02E-06	ARL15	--<br>HSPB3	--<br>+201393	I,II
H3GA0046586	16	46700580	1.22E-05	--	PIK3R1<br>--	-176971<br>--	I,II
ASGA0073321	16	46841735	1.30E-05	--	PIK3R1<br>SLC30A5	-318126<br>+443025	I,II
MARC0029744	16	47868335	9.08E-06	BDP1	U6<br>MCCC2	-235317<br>+48956	I,II

The method numbers I, II, and III represent the FarmCPU, GLM, and MLM, respectively. The black bold fonts represent significant loci.

Supplementary Table S2

Format: DOC

Title:Potential SNP in genome-wide association analysis of MOT.

SNP	Chr	Location （bp）	P-value	Gene names	Bilateral genes	Distance	Method
ALGA0122208	1	2600235	1.36E-05	BRINP1	--	--	I,II
					CDK5RAP2	+443389	
ASGA0031806	7	20987924	1.55E-05	SLC17A4	H2BC7	-89721	I,II
					BTN1A1	+2854	
ALGA0039452	7	22008244	5.74E-06	--	ZKSCAN8	-7425	I,II
					ZSCAN9	73927	
ALGA0039459	7	22038961	5.74E-06	--	ZNF389	-26989	I,II
					ZSCAN9	+43210	
ASGA0031860	7	22075114	5.74E-06	--	ZSCAN9	-7057	I,II
					ZKSCAN4	+17945	
WU_10.2_16_56105261	16	51735901	3.06E-05	--	NEURL1B	-154775	I,II
					U6	+163771	

The method numbers I, II, and III represent the FarmCPU, GLM, and MLM, respectively.

Supplementary Table S3

Format: DOC

Title:Potential SNP in genome-wide association analysis of ABN.

SNP	Chr	Location（bp）	P-value	Gene names	Bilateral genes	Distance	Method
MARC0054558	8	84708131	2.92E-05	USP38	GAB1<br>--	-316510<br>--	I,II
ALGA0122094	8	84730285	2.92E-05	USP38	GAB1<br>--	-338664<br>--	I,II

The method numbers I, II, and III represent the FarmCPU, GLM, and MLM, respectively.

Supplementary Table S4

Format: DOC

Title:Potential SNP in genome-wide association analysis of MMP.

SNP	Chr	Location （bp）	P-value	Gene names	Bilateral genes	Distance	Method
MARC0029602	4	15778435	3.10E-06	TMEM65	FAM91A1	-96609	Ⅰ,II,III
					ANXA13	+11806	
H3GA0039852	14	38703569	1.41E-05	--	RITA1	-29203	Ⅰ,II
					IQCD	+5257	
ALGA0077075	14	38841343	2.88E-06	--	OAS1	-10575	I,II
					RPH3A	25756	
MARC0080850	14	38870474	1.41E-05	--	OAS1	-11065	I,II
					PTPN11	+331695	
ASGA0062900	14	38886155	1.41E-05	--	OAS1	-26746	I,II
					PTPN11	316014	
ASGA0062901	14	38907084	1.41E-05	--	OAS1	-47675	I,II
					PTPN11	295085	
MARC0027505	14	38927125	1.41E-05	--	OAS1	-67716	I,II
					PTPN11	275044	
ASGA0062984	14	41455034	5.60E-06	ANKRD13A	UBE3B	-12558	I,II
					MYO1H	+15882	
MARC0016119	14	41594250	1.10E-05	GLTP	MYO1H	-34805	I,II
					ACACB	27324	
ALGA0077164	14	41747992	8.34E-06	MVK	ACACB	-8076	I,II

ALGA0077177	14	41961691	3.60E-06	KCTD10	ALKBH2<br>SSH1	12513<br>-6775	I,II
ALGA0077178	14	41985970	8.34E-06	MYO1H	CORO1C<br>DAO<br>CORO1C	+101202<br>-35337<br>76923	I,II

Supplementary Table S5

Format: DOC

Title:Potential SNP in genome-wide association analysis of AIR.

SNP	Chr	Location （bp）	P-value	Gene names	Bilateral genes	Distance	Method
WU_10.2_6_43634682	14	132324567	9.40E-06	--	PSP-II	-42839	I,II
					C14H10orf120	+21103	
MARC0041976	14	29797361	1.69E-05	ATP6V0A2	PITPNM2	-2920	I,II
					ARL6IP4	+88681	
WU_10.2_14_143928481	14	132481394	8.65E-06	PLEKHA1	C14H10orf120	-133823	I,II
					PSTK	+50841	
WU_10.2_14_143932451	14	132477432	1.01E-05	PLEKHA1	C14H10orf120	-129861	I,II
					PSTK	54803	
WU_10.2_14_144068279	14	132481745	8.65E-06	PLEKHA1	C14H10orf120	-134174	I,II
					PSTK	50490	

The method numbers I, II, and III represent the FarmCPU, GLM, and MLM, respectively.

Supplementary Table S6

Format: DOC

Title:Potential SNP in genome-wide association analysis of ROS.

SNP	Chr	Location （bp）	P-value	Gene names	Bilateral genes	Distance	Method
H3GA0033068	12	6322520	1.35E-05	LLGL2	HID1	-11572	I,II
					OTOP3	+440	
WU_10.2_7_10722135	7	10289420	2.94E-05	GFOD1	RNF182	-33566	III
					CD83	+69791	

The method numbers I, II, and III represent the FarmCPU, GLM, and MLM, respectively.
